# An adenocarcinoma in an inverted Meckel’s diverticulum with intussusception

**DOI:** 10.1186/s40792-023-01680-1

**Published:** 2023-06-05

**Authors:** Yamato Miwa, Yo Sato, Kenichiro Hirano, Eiji Sunami, Motoko Takahashi, Shin-ichi Kosugi, Takeshi Suda, Go Hasegawa

**Affiliations:** 1 Department of Digestive and General Surgery, Uonuma Kikan Hospital, 4132 Urasa, Minami-Uonuma, Niigata 949-7302 Japan; 2 Department of Gastroenterology and Hepatology, Uonuma Kikan Hospital, 4132 Urasa, Minami-Uonuma, Niigata 949-7302 Japan; 3 Department of Pathology, Uonuma Kikan Hospital, 4132 Urasa, Minami-Uonuma, Niigata 949-7302 Japan

**Keywords:** Inverted Meckel's diverticulum, Adenocarcinoma, Intussusception

## Abstract

**Background:**

Adenocarcinoma in an inverted Meckel's diverticulum with intussusception has not been reported to date. We discuss the clinical issues concerning this rare condition and review the relevant literature.

**Case presentation:**

A 71-year-old Japanese female was referred to our hospital for further investigation of severe anemia. Computed tomography revealed a tumorous lesion in the terminal ileum. Capsule endoscopy did not provide detailed images. Exploratory laparoscopy revealed intussusception in the terminal ileum. An intraluminal tumor 70 cm proximal to the ileocecal valve was observed to be the lead point. Partial resection including the tumor was performed. Macroscopically, a polypoid tumor at the tip of an inverted diverticulum-like structure was observed. The tumor was histologically composed of adenocarcinoma accompanied by gastric and pyloric gland metaplasia in the background mucosa, which was confirmed by immunohistochemical staining. Based on these characteristics, this tumor is considered to have developed from the ectopic gastric mucosa in a Meckel's diverticulum.

**Conclusions:**

When we encounter patients with unfamiliar lesions in the small bowel, we need to differentiate Meckel's diverticulum related disease. Meckel's diverticulum can invert into the lumen of the small bowel and cause an intussusception, and has potential of malignant transformation.

## Background

Meckel's diverticulum (MD) is a remnant of the omphalomesenteric duct and is a true congenital diverticulum located in the antimesenteric surface of the ileum. It is often approx. 2 feet from the ileocecal valve has been observed in ~ 2% of the total population [1]. The common complications of MD include intestinal obstruction, intussusceptions, diverticulitis, volvulus, and gastrointestinal bleeding. It has also been reported that MD can be inverted into the lumen of the ileum, which causes abdominal pain and anemia [2]. Although most MD-related diseases are observed incidentally and are benign in nature, malignant neoplasms occur in ~ 0.5–3.2% of MD cases [3].

We herein report a case of a small bowel tumor that was discovered during a detailed examination for anemia. The tumor was intraoperatively observed to serve as a lead point of an intussusception and was pathologically diagnosed as adenocarcinoma at the tip of the inverted MD. We also discuss the clinical issues concerning this rare condition and review the relevant literature.

## Case presentation

A 71-year-old Japanese female had visited another hospital with the complaints of general fatigue and severe anemia, with a 3.5-g/dL hemoglobin level that was detected by laboratory examination. After the patient was administered a 1680-mL red blood cell transfusion, she was referred to our hospital's Department of Hematology for a further investigation of her anemia. Her medical history included appendectomy, hysterectomy for myoma, left hemithyroidectomy for follicular adenoma, and Alzheimer-type dementia. She had also undergone a distal gastrectomy with cholecystectomy for stomach cancer and gallstone 15 years earlier. She was a habitual smoker and alcohol consumer. She had no other symptoms at presentation, including fever, body weight loss, abdominal pain, change in bowel habit, and melena. No specific findings were observed in the physical examination, with neither abdominal distention nor a palpable tumor. Laboratory examinations showed a red blood cell count at 350 × 10^4^ /μL and hemoglobin level at 9.3 g/dL. The mean corpuscular volume (MCV), 85.1 fL, was within normal limits, and the mean corpuscular hemoglobin (MCH) value, 26.6 pg, and mean corpuscular hemoglobin concentration (MCHC) at 31.2% were slightly lower than the normal limits. The patient's serum iron and vitamin B12 levels were 34 µg/dL and 294 pg/mL, respectively. The serum carcinoembryonic antigen (CEA) and carbohydrate antigen (CA) 19-9 levels were within normal limits, whereas the soluble interleukin-2 receptor level at 538 U/mL was slightly higher than the normal limit (range 122–496 U/mL).

**Fig. 1:**
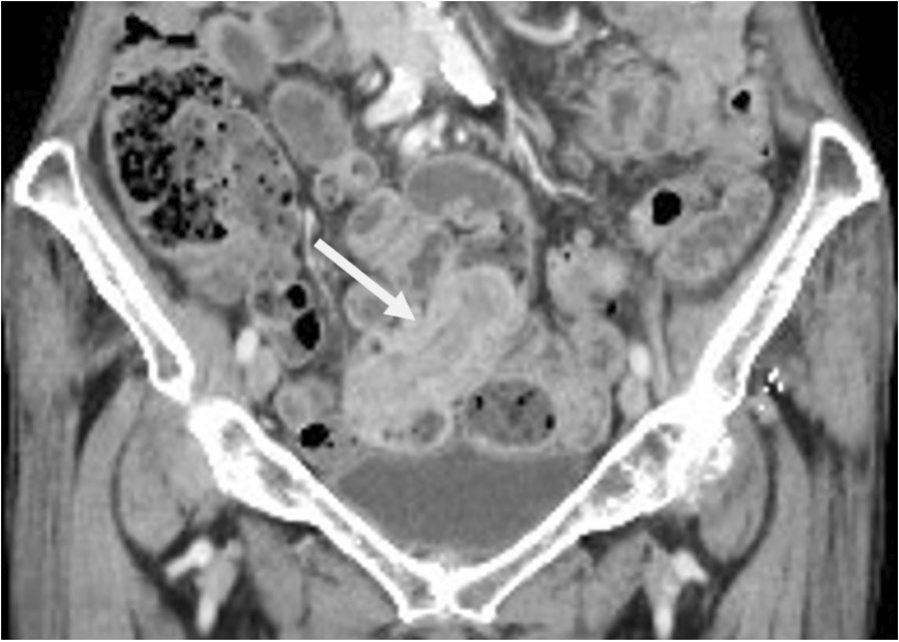
CT showed a tumorous lesion with contrast enhancement in the terminal ileum (arrow)

**Fig. 2:**
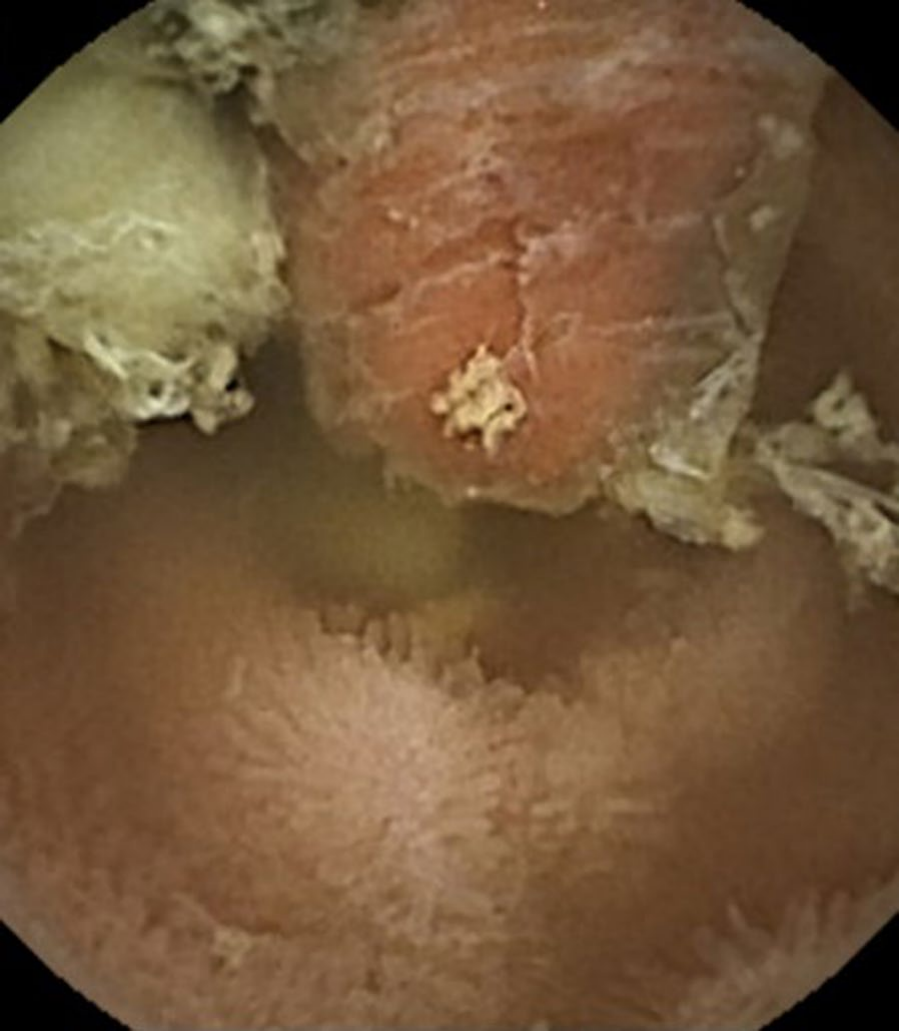
Capsule endoscopy revealed a polypoid lesion with erythematous and irregular mucosa in the terminal ileum, but it could not provide detailed images

A diagnosis of iron deficiency anemia was made, and oral iron therapy was commenced. Screening computed tomography (CT) for gastrointestinal bleeding of obscure origin demonstrated a tumorous lesion with contrast enhancement in the terminal ileum, with the proximal small intestine slightly dilated (Fig. 1). Capsule endoscopy showed a polypoid lesion with erythematous and irregular mucosa in the terminal ileum. Temporary capsule retention was observed, but detailed images could not be obtained (Fig. 2). Based on these clinical and radiological findings, an ileal tumor was considered as a possible cause of the patient's occult hemorrhage and anemia, and she was referred to our department for surgical intervention.

**Fig. 3:**
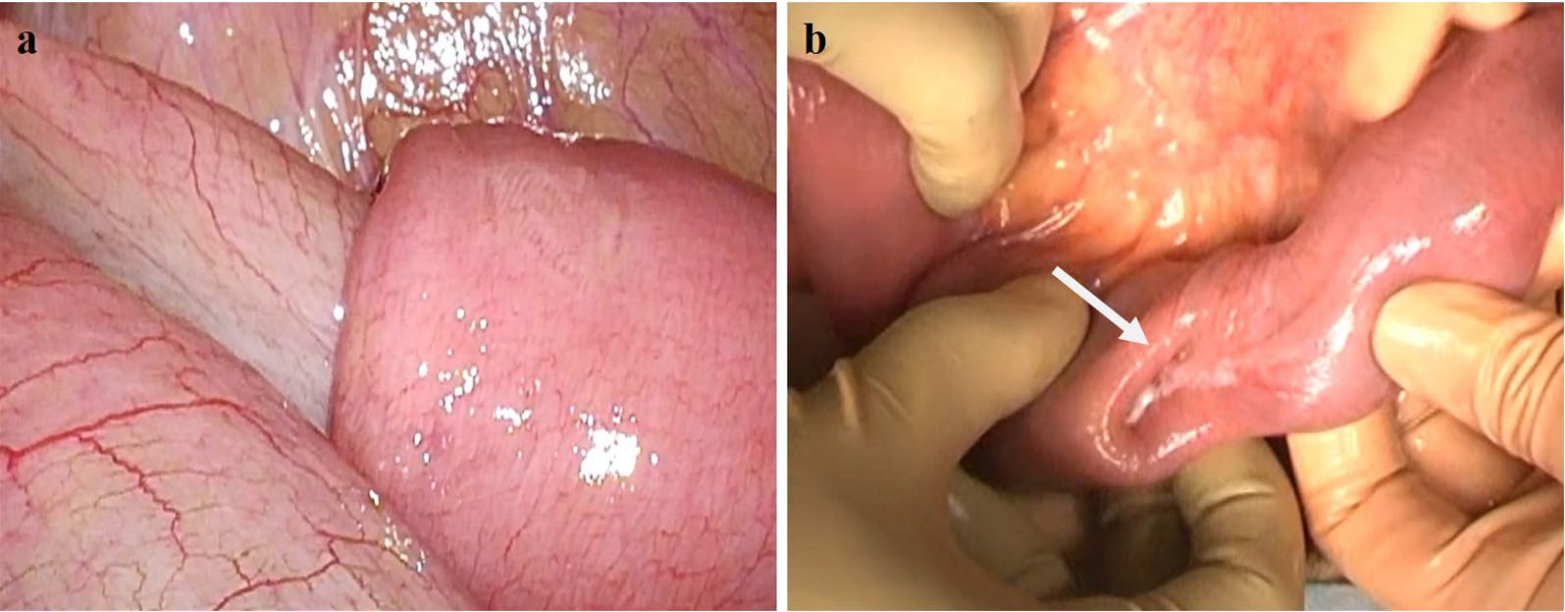
Intraoperative findings. **a:** Laparoscopy showed an intussusception in the terminal ileum. **b:** The lead point was an intraluminal tumor with a serosal pit (arrow), which was 70 cm proximal to the ileocecal valve

Exploratory laparoscopy revealed an intussusception in the terminal ileum (Fig. 3a), which was drawn through the mini-laparotomy wound and reduced by using Hutchinson's maneuver. The lead point was an intraluminal tumor with a serosal pit 70 cm proximal to the ileocecal valve (Fig. 3b). A partial resection of the ileum including the tumor was thus performed.

**Fig. 4:**
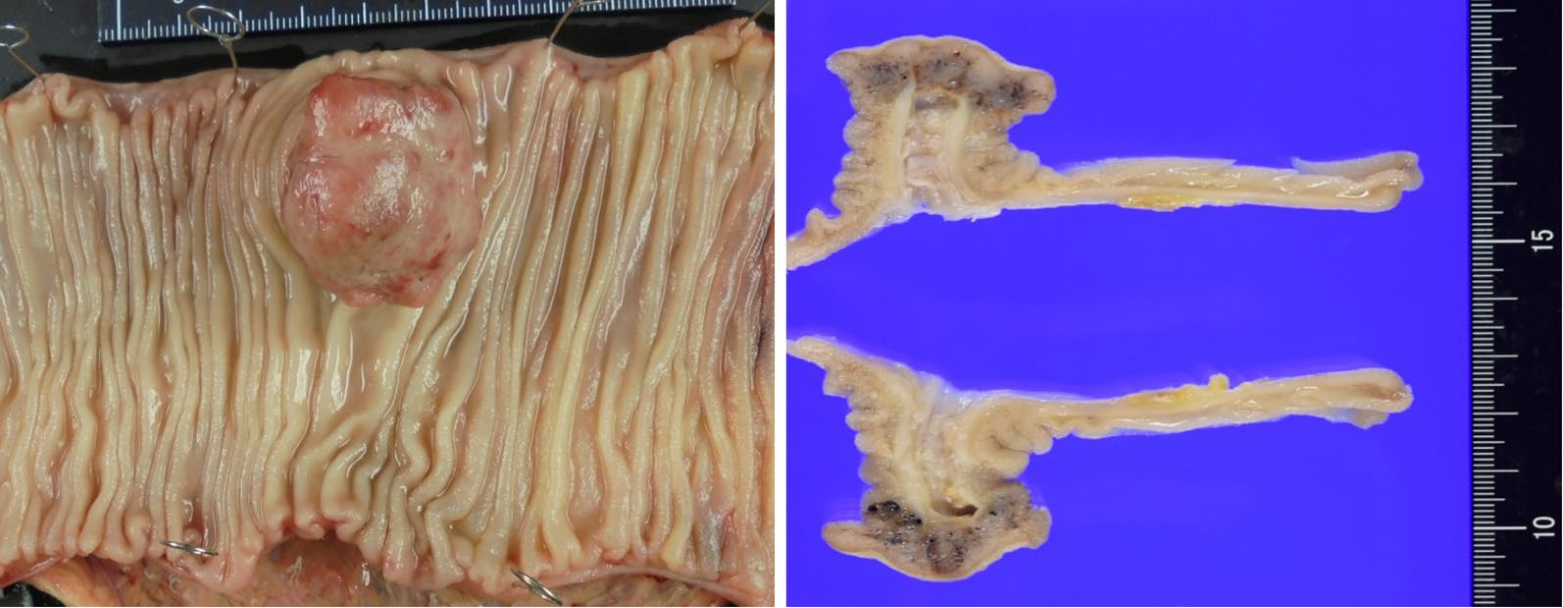
The macroscopic examination of the resected specimen revealed a polypoid tumor at the tip of an inverted diverticulum-like structure. The max. tumor dia. was 35 mm

**Fig. 5:**
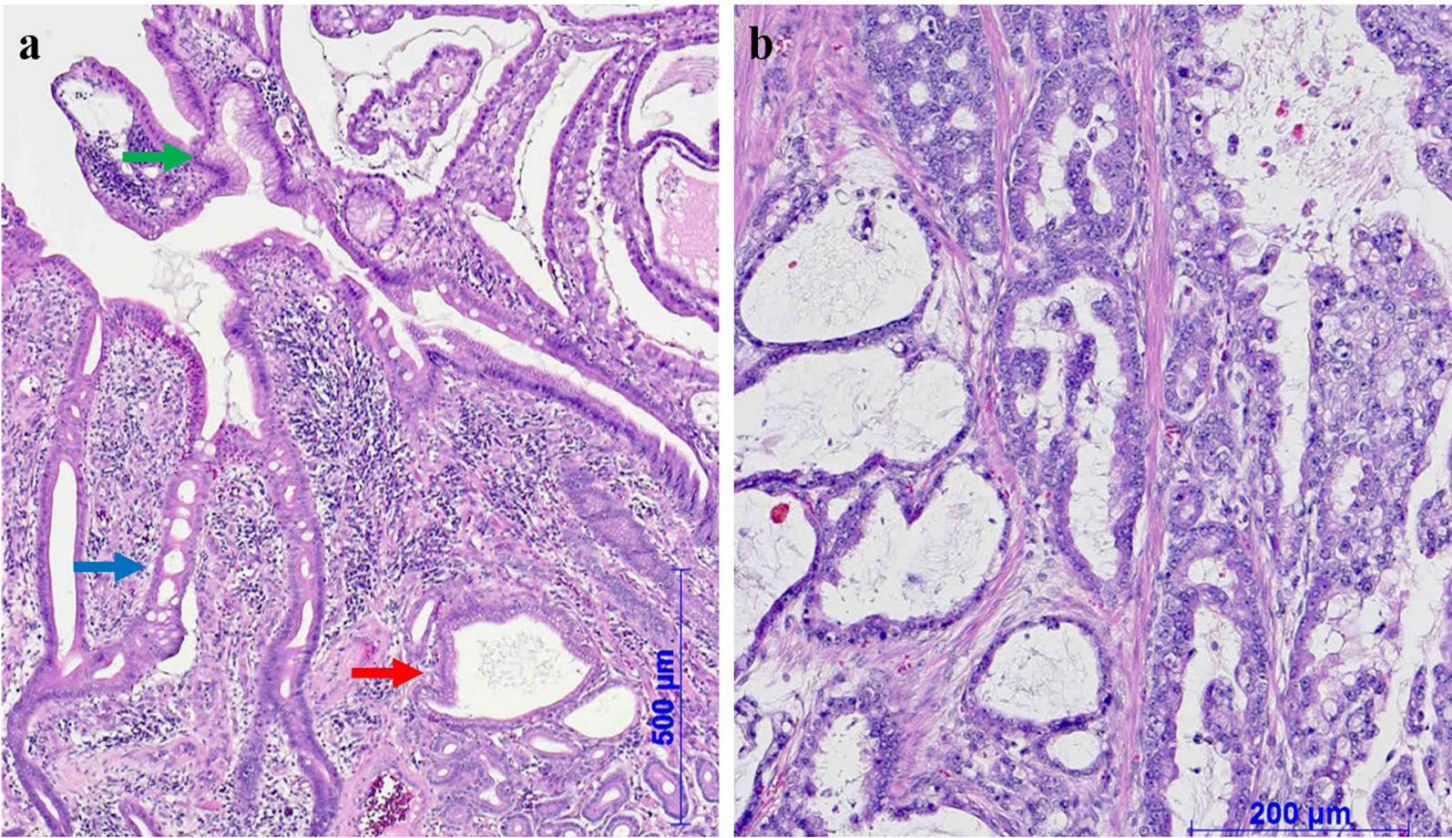
Histological examination of the inverted diverticulum-like structure by hematoxylin–eosin staining. **a** The intestinal epithelium (blue arrow) was mixed with pyloric glands (red arrow) and gastric foveolar epithelium (green arrow). **b** A well-to-moderately differentiated adenocarcinoma was adjacent to the background ectopic gastric mucosa

**Fig. 6:**
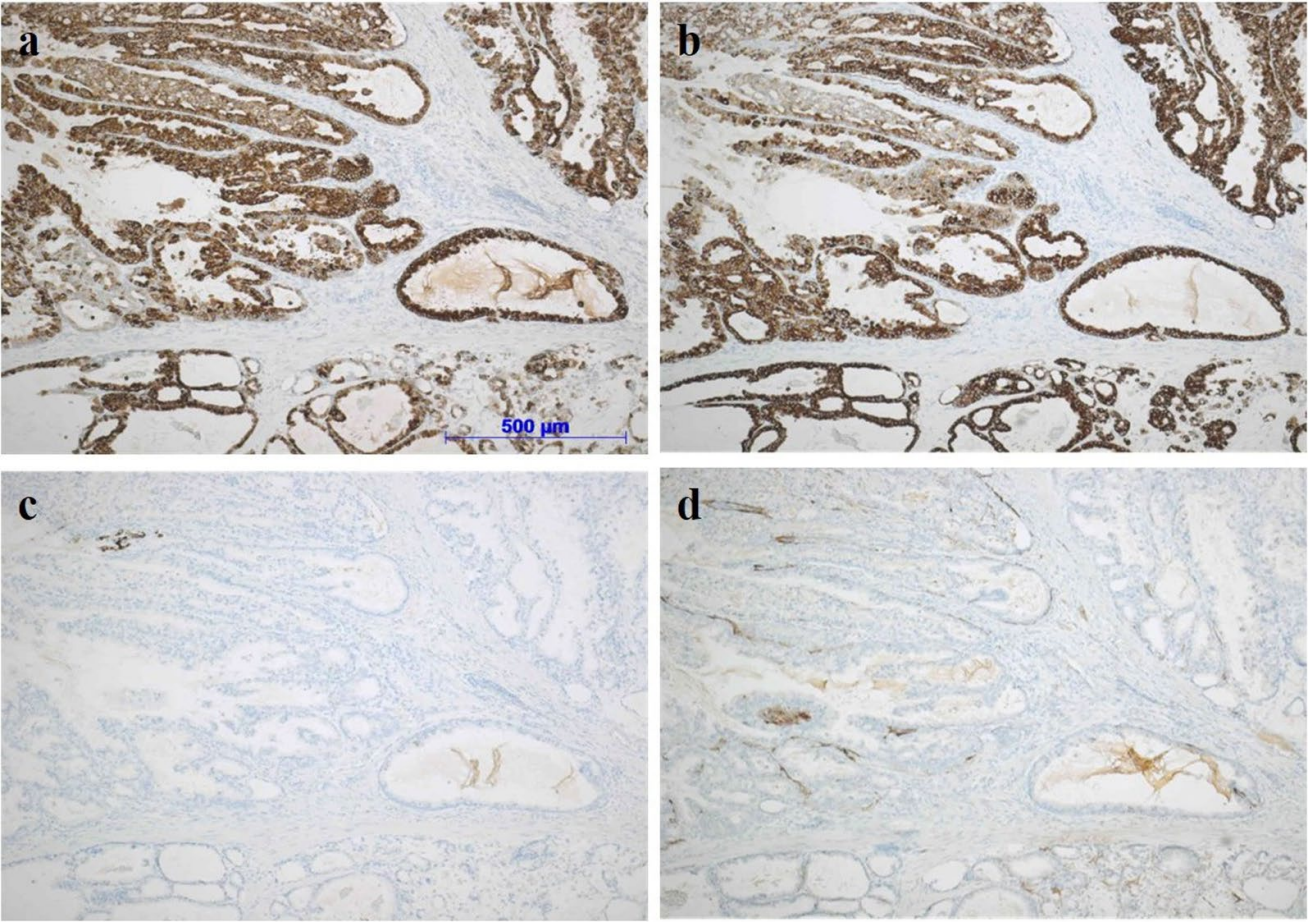
Immunohistochemical staining revealed that the glands in the tumor was positive for mucin core protein (MUC)5AC (**a**) and MUC6 (**b**), but negative for MUC2 (**c**) and CD10 (**d**)

The macroscopic examination of the resected specimen revealed a polypoid tumor at the tip of an inverted diverticulum-like structure (Fig. 4). The tumor was histologically composed of well-to-moderately differentiated adenocarcinoma with subserosal invasion, accompanied by the ectopic gastric mucosa (Fig. 5). No metastatic lymph nodes were observed in the resected mesenterium. Immunohistochemical staining revealed that the glands in the tumor was positive for mucin core protein (MUC)5AC and MUC6, but negative for MUC2 and CD10 (Fig. 6a–d). Based on these macroscopic and microscopic characteristics, we considered the tumor a completely gastric-type adenocarcinoma arising from the ectopic gastric mucosa in the Meckel's diverticulum.

The postoperative course was uneventful, and the patient was discharged from our hospital on day 7. She has been on the close follow-up course due to the scarce evidence of adjuvant therapy for this disease.

## Discussion

Meckel’s diverticulum (MD) has been reported to invert into the ileal lumen; such cases are referred as an 'inverted MD' and can cause gastrointestinal bleeding, ulceration, small bowel obstruction, and/or intussusception [2]. The possible causes of this inversion are as follows: (1) intestinal peristalsis in the segment of bowel proximal to the MD, (2) movability of the tip of an unfixed MD to the mesentery or the intestine, and (3) negative intraluminal pressure which occurs with the passage of intestinal contents [4–6]. Due to it rare nature, inverted MD is difficult to diagnose on imaging. Some specific findings on CT scans are subserous adipose tissue that has been pulled into the intestinal lumen as a central fat density area, surrounded by soft tissue with the same density as the intestinal wall [7], or an intraluminal polypoid lesion in the small intestine covered with a thick collar of enhancing soft tissue [8].

Although about half of the cases of inverted MD have been reported to be related to the tumor that increases the chance of intussusception [9], the tumorous lesion observed on our patient's CT scans is retrospectively considered an inverted MD itself rather than the tumor at the tip of the MD. Proximal dilatation of the small bowel without intussusception might imply a chronic and low-grade obstruction.

The frequency of an MD associated with a tumor has been estimated as 3–6% of MD cases [10]. Carcinoid is the most common malignant neoplasm developed in MD, accounting for 33–44% of MD cases, followed by leiomyosarcoma at 18–25%, adenocarcinoma at 12–16%, and gastrointestinal stromal tumor (GIST) at 12% [3]. Although the clinical symptoms and signs of adenocarcinoma in MD are various, such as gastrointestinal bleeding [11], elevation of serum CEA and/or CA19-9 levels [12, 13], and spontaneous rupture [14], we have found no published report of adenocarcinoma in an inverted MD with intussusception. MDs often contains heterotopic tissue such as pancreatic tissue or gastric, duodenal, jejunal, and/or colonic mucosa, which are involved in the carcinogenesis [15]. Immunostaining using cytokeratin or mucin core protein (MUC) is considered useful for inferring the origin of the malignancy [16].

Considering that MUC5AC and MUC6 are usually expressed in the gastric foveolar epithelium and the pyloric gland of the stomach, respectively, whereas MUC2 and CD10 are usually expressed in the small intestinal epithelium, we suspect that our patient's adenocarcinoma developed from the ectopic gastric mucosa of the MD. No causative factors have been identified for the malignant transformation of the ectopic gastric mucosa, but it has been proposed that such a transformation may have more malignant potential than normal intestinal mucosa, partly explained by a sustained chronic inflammation due to Helicobacter pylori infection in the diverticulum [3].

It is extremely difficult to diagnose malignancies in an MD preoperatively [10]. Capsule endoscopy and balloon-assisted enteroscopy are useful for the preoperative diagnosis of malignant tumors in an MD [11, 17]. The capsule endoscopy performed for our patient revealed a suspected polypoid lesion protruding into the intestinal lumen in the terminal ileum, but detailed images could not be obtained. Although the diagnostic accuracy of capsule endoscopy for inverted MDs is unknown because of the limited data [2], in our patient's case it seemed difficult for the capsule endoscopy to capture the whole image when the MD was inverted in the isoperistaltic direction as shown in the CT scan. It was likely that the capsule endoscopy passed over the inverted MD and overlooked the adenocarcinoma at the tip of the diverticulum. We also initially misidentified the polypoid lesion as a submucosal tumor such as a gastrointestinal stromal tumor (GIST) because the surface seemed to be covered with non-neoplastic but inflammatory intestinal mucosa.

Retrograde double-balloon enteroscopy may be able to capture an entire image of an inverted MD, and the findings in such cases were reported to be a submucosal tumor-like lesion exhibiting intestinal villous mucosa on the surface at the head and Kerckring's folds at the stalk of the antimesenteric attachment [18]. Because our patient's general condition did not necessitate an emergency intervention, we should have considered performing retrograde double-balloon enteroscopy, as its findings might have enabled a definitive diagnosis of both the inverted MD and the associated adenocarcinoma simultaneously.

Standard treatments for carcinoma of an MD are not yet established, and the significance of prophylactic lymph node dissection or adjuvant therapy remains unknown. Regardless of the operative approach, a complete resection with no residual tumor is mandatory.

## Conclusions

Preoperative diagnoses of various diseases related to MD are generally difficult, but clinicians should recognize that an MD can invert into the lumen of the small bowel and cause an intussusception, and such cases often have heterotopic tissue with a potential of malignant transformation. When patients with unfamiliar lesions in the distal small bowel are encountered, a close examination should be performed with an awareness of potential MD-related disease.

## Data Availability

All data generated or analyzed during this study are included in this published article.

## Abbreviations

MD: Meckel's diverticulum
MCV: Mean corpuscular volume
MCH: Mean corpuscular hemoglobin
MCHC: Mean corpuscular hemoglobin
CEA: Carcinoembryonic antigenand
CA: Carbohydrate antigen
CT: Computed tomography
MUC: Mucin core protein
